# Supplementary material for: Transgenic ferret models define pulmonary ionocyte diversity and function
Source: Nature. 2023 Sep 20;621(7980):857–67. doi: 10.1038/s41586-023-06549-9 (PMC10533402; doi:10.1038/s41586-023-06549-9)
Supplement: Supplementary file 1 — Supplementary Fig. 1. Raw data gels. Kidney tissue lysates from FOXI1-KO and WT animals were collected and subjected to western blotting with FOXI1 antibody. Uncropped images of western blots. Kidney tissue lysates from FOXI1-KO and WT animals were collected and subjected to western blotting with FOXI1 antibody. n = 2 ferret donors in WT group; n = 3 ferret donors in FOXI1-KO group. Supplementary Fig. 2. Gating strategy to sort FOXI1-lineage (EGFP-positive) cells from non-traced epithelial cells. Gating strategy to sort FOXI1-lineage (EGFP-positive) cells from non-traced epithelial cells. ALI cultures from ROSA-TG::FOXI1-CreERT2 ferrets were induced with 4-hydroxytamoxifen to label FOXI1-lineage (EGFP-positive) cells. [file 41586_2023_6549_MOESM1_ESM.pdf]

nature portfolio

https://doi.org/10.1038/s41586-023-06549-9

Supplementary information

Transgenic ferret models define pulmonary ionocyte diversity and function

In the format provided by the authors and unedited

Supplementary Information Figure 1: Raw Data Gels

WT: WT ferret Kidney lysate; KO: *FOXI1*-KO ferret Kidney lysate; B: Blank

Device: Biorad WB
Gel: 8% Gel
Denature: 95°C, 5 mins
Electrophoresis:
80V, 20 mins; 100V, 1hr
Transfer:
200 mA, 90 mins, on ice

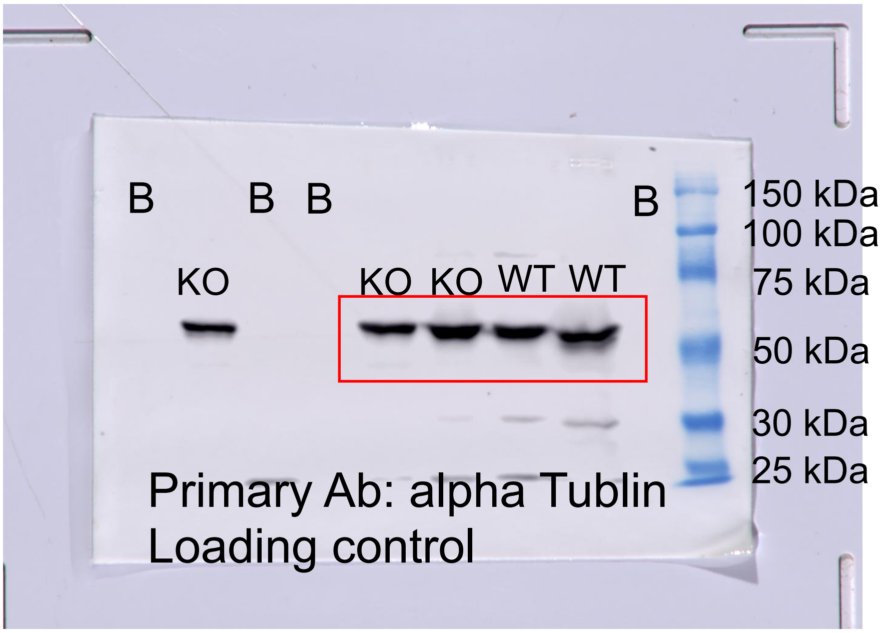

Controls were run on the same gel as loading control. Red rectangle indicates how gels were cropped for final figure.

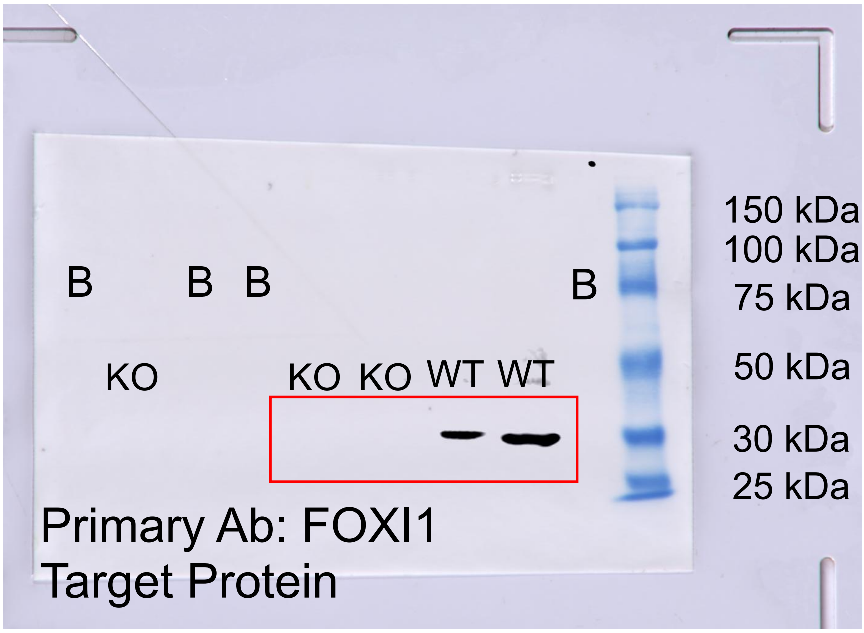

SI Figure 1: Uncropped images of Western blots. Kidney tissue lysates from *FOXI1*-KO and WT animals were collected and subjected to Western blotting with FOXI1 antibody. n = 2 ferret donors in WT group; n = 3 ferret donors in *FOXI1*-KO group.

Supplementary Information Figure 2: *FOXI1*-lineage cells enrichment gate strategy

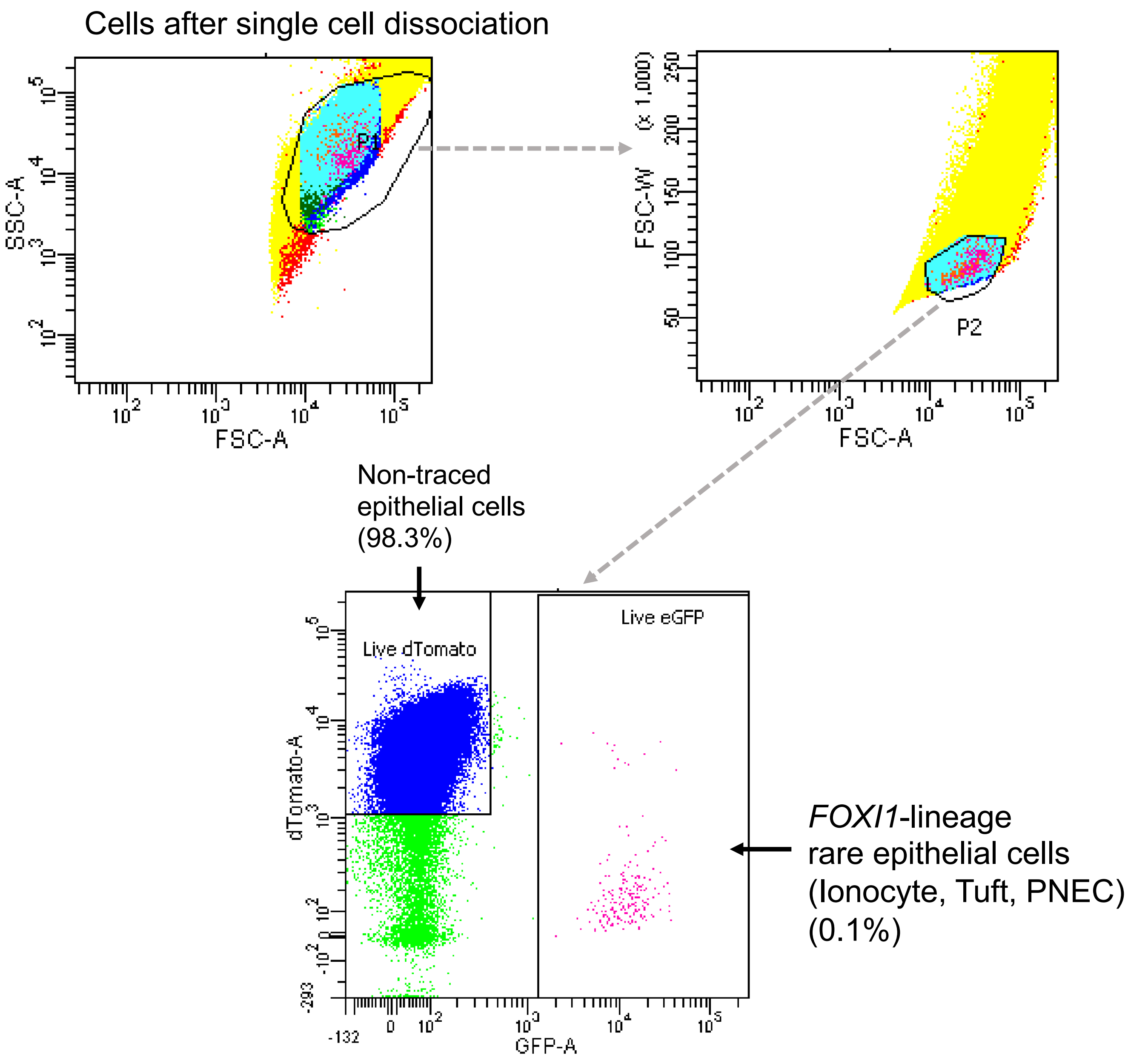

SI Figure 2: Gating strategy to sort *FOXI1*-lineage (eGFP positive) cells from non-traced epithelial cells. ALI cultures were from ROSA TG:: *FOXI1*-Cre^{ERT2} ferrets and *FOXI1*-lineage (eGFP positive) cells were induced with hydroxytamoxifen.
