# Supplementary material for: Transgenic ferret models define pulmonary ionocyte diversity and function
Source: Nature. 2023 Sep 20;621(7980):857–67. doi: 10.1038/s41586-023-06549-9 (PMC10533402; doi:10.1038/s41586-023-06549-9)
Supplement: Supplementary file 2 — Reporting Summary [file 41586_2023_6549_MOESM2_ESM.pdf]

nature portfolio

Corresponding author(s): John F. Engelhardt

Last updated by author(s): 8/8/2023

Reporting Summary

Nature Portfolio wishes to improve the reproducibility of the work that we publish. This form provides structure for consistency and transparency in reporting. For further information on Nature Portfolio policies, see our Editorial Policies and the Editorial Policy Checklist.

Statistics

For all statistical analyses, confirm that the following items are present in the figure legend, table legend, main text, or Methods section.

n/a	Confirmed	
☐	☒	The exact sample size (*n*) for each experimental group/condition, given as a discrete number and unit of measurement
☐	☒	A statement on whether measurements were taken from distinct samples or whether the same sample was measured repeatedly
☐	☒	The statistical test(s) used AND whether they are one- or two-sided<br>*Only common tests should be described solely by name; describe more complex techniques in the Methods section.*
☐	☒	A description of all covariates tested
☐	☒	A description of any assumptions or corrections, such as tests of normality and adjustment for multiple comparisons
☐	☒	A full description of the statistical parameters including central tendency (e.g. means) or other basic estimates (e.g. regression coefficient) AND variation (e.g. standard deviation) or associated estimates of uncertainty (e.g. confidence intervals)
☐	☒	For null hypothesis testing, the test statistic (e.g. *F*, *t*, *r*) with confidence intervals, effect sizes, degrees of freedom and *P* value noted<br>*Give P values as exact values whenever suitable.*
☐	☒	For Bayesian analysis, information on the choice of priors and Markov chain Monte Carlo settings
☐	☒	For hierarchical and complex designs, identification of the appropriate level for tests and full reporting of outcomes
☐	☒	Estimates of effect sizes (e.g. Cohen's *d*, Pearson's *r*), indicating how they were calculated

Our web collection on statistics for biologists contains articles on many of the points above.

Software and code

Policy information about availability of computer code

Data collection	Zen software (Immunofluorescence imaging collection): version 2.3; Acquire and Analyze 2.3 (electrophysiology data collection); Novaseq control software v1.7.5 (scRNA seq data collection); Bio-Rad Real-Time PCR (Relative gene expression data collection): version 2.3; Zeiss software FRAP (airway viscosity data collection): version 2.3.
Data analysis	Zen software (Immunofluorescence imaging analysis): version 2.3; Acquire and Analyze 2.3 (electrophysiology data analysis); Metamorph (imaging analysis): version 7.0; Ingenuity Pathway Analysis (IPA): version 01-21-03; GraphPad Prism 9; R 4.2.0; Image J: version 2.3.0/1.53q; PMOD (Mucociliary Clearance analysis): version 4.2; Bio-Rad Real-Time PCR: version 2.3; Zeiss software FRAP: version 2.3; BD FACSDiva 8.0.1 Software; Kallisto toolkit: version 0.48; DropletUtils package: version 1.20.0; R package 'brms': version 2.17.0, MAST: version 1.22.0; ElPiGraph.R: version 1.0.0, R package “prop.test”: version 3.6.2. R markdown scripts are available upon request.<br>Customized code for the computation of cross-species similarity between rare cells is available on GitHub (https://github.com/yifand64/ferret_ionocyte).

For manuscripts utilizing custom algorithms or software that are central to the research but not yet described in published literature, software must be made available to editors and reviewers. We strongly encourage code deposition in a community repository (e.g. GitHub). See the Nature Portfolio guidelines for submitting code & software for further information.

Data

Policy information about availability of data

All manuscripts must include a data availability statement. This statement should provide the following information, where applicable:

- Accession codes, unique identifiers, or web links for publicly available datasets
- A description of any restrictions on data availability
- For clinical datasets or third party data, please ensure that the statement adheres to our policy

Single-cell sequencing data is available in GEO, accession (GSE233654): https://www.ncbi.nlm.nih.gov/geo/query/acc.cgi?acc=GSE233654.
Data for Figures and Extended Data Figures are available as Source Data.
The publicly available Genome assembly MusPutFur1.0 (https://www.ncbi.nlm.nih.gov/datasets/genome/GCF_000215625.1/) was used as ferret reference genome in this study. The publicly available Genome assembly GRCh38.p13 (https://www.ncbi.nlm.nih.gov/datasets/genome/GCF_000001405.39/) was used for identification of unannotated ferret genes by using human orthologs. The publicly available Genome assembly GRCm39 (https://www.ncbi.nlm.nih.gov/datasets/genome/GCF_000001635.27/) was used for identification of unannotated ferret genes by using mouse orthologs.

Human research participants

Policy information about studies involving human research participants and Sex and Gender in Research.

Reporting on sex and gender	None
Population characteristics	None
Recruitment	None
Ethics oversight	None

Note that full information on the approval of the study protocol must also be provided in the manuscript.

Field-specific reporting

Please select the one below that is the best fit for your research. If you are not sure, read the appropriate sections before making your selection.

☒ Life sciences ☐ Behavioural & social sciences ☐ Ecological, evolutionary & environmental sciences

For a reference copy of the document with all sections, see nature.com/documents/nr-reporting-summary-flat.pdf

Life sciences study design

All studies must disclose on these points even when the disclosure is negative.

Sample size	No predetermined sample size calculation was performed in this study. Based on data we collected, we think the number of samples in each group were adequate to confirm our findings. Regarding to scRNA-seq experiments, we have excellent cell diversity coverage of ferret epithelial cell of interest and already have shown in our study that all cells are well covered by each ferret donor in each group. Given the complexity, size and reproducibility within each figures, we felt that the number of samples shown was appropriate to address the questions in this study.
Data exclusions	No data were excluded from analysis.
Replication	All attempts of replication were successful as shown in our Figures.
Randomization	Samples/organisms were allocated into experimental groups based on genotype.
Blinding	Blinding was not necessary in this study since assays used unbiased quantification methods.

Reporting for specific materials, systems and methods

We require information from authors about some types of materials, experimental systems and methods used in many studies. Here, indicate whether each material, system or method listed is relevant to your study. If you are not sure if a list item applies to your research, read the appropriate section before selecting a response.

Materials & experimental systems

n/a	Involved in the study	
☐	☒	Antibodies
☐	☒	Eukaryotic cell lines
☒	☐	Palaeontology and archaeology
☐	☒	Animals and other organisms
☒	☐	Clinical data
☒	☐	Dual use research of concern

Methods

n/a	Involved in the study	
☒	☐	ChIP-seq
☐	☒	Flow cytometry
☒	☐	MRI-based neuroimaging

Antibodies

Antibodies used

Primary Antibodies Used (dilution factor; catalogue number; company):
anti NKA (1/100-1/300; a5, DSHB UIOWA, dshb.biology.uiowa.edu)
anti CFTR (1/100-1/300; CFTR antibody 596, cftrantibodies.web.unc.edu)
anti-Keratin 5 (1/500; 905501, Biolegend)
anti-TRPM 5 (1/300; ACC-045, Alamone)
anti-SYP (1/200; sc-17750, Santa Cruz Biotechnology)
anti-acetylated Tublin (1/1000, T7451, Sigma Aldrich)
anti-ATP6V1G3 (1/500, HPA028701, Sigma Aldrich)
anti-Ki-67 (1/500, 14-5698-82, eBioscience)
anti-BSND (1/500; ab196017, Abcam)
anti-FOXI1 (1/500; ab20454, Abcam)
anti-EGFP (1/300; ab13970, Abcam)
anti-p63 (1/300; Clone Poly6190, Stemcell technology)
anti-Muc5B (1/300; HPA008246, Sigma)
anti-Muc5AC (1/300; ab3649, Abcam)
Secondary Antibodies Used (dilution factor; catalogue number; company):
Alexa Fluor 647 donkey anti-mouse IgG (1/250, A31571, Molecular Probes)
Alexa Fluor 488 donkey anti-goat IgG (1/250, A11055, Invitrogen)
Alexa Fluor 488 donkey anti-chicken IgG (1/250, 703-546-155, Jackson ImmunoResearch)
Alexa Fluor 488 donkey anti-rabbit IgG (1/250, A21206 Invitrogen)
Alexa Fluor 568 donkey antigoat IgG (1/250, A-11057, Jackson ImmunoResearch)
Alexa Fluor 647 donkey Anti-Rabbit IgG (1/250,711-606- 152, Jackson ImmunoResearch)
Alexa Fluro 555 donkey Anti-mouse IgG (1/250, A31570, Life Technologies).

Validation

anti-NKA (Confirmed Species reactivity: Avian, Drosophila, Fish, Frog, Honeybee, Human, Insect, Mackerel, Mammal, Mosquito, Zebrafish; Applications: FFPE, Immunofluorescence, Immunohistochemistry, Immunoprecipitation, Western Blot; Validation: Manufacturer - https://dshb.biology.uiowa.edu/a5)
anti-CFTR (Confirmed Species reactivity: Human; Applications: Immunofluorescence, Western Blot; Validation: Manufacturer - https://cftrantibodies.web.unc.edu/cell-staining/; https://cftrantibodies.web.unc.edu/western-blot/)
anti-Keratin 5 (Confirmed Species reactivity: Human; Applications: Immunohistochemistry; Validation: Manufacturer - https://www.biolegend.com/Files/Images/media_assets/pro_detail/datasheets/905501-keratin-5-polyclonal-antibody-purified-10956-IFU-Rev-7.pdf?v=20220914082417)
anti-TRPM 5 (Confirmed Species reactivity: Human, mouse, rat; Applications: Immunofluorescence, Western Blot, Immunohistochemistry; Validation: Manufacturer - https://www.alomone.com/p/anti-trpm5/ACC-045)
anti-SYP (Confirmed Species reactivity: Human, mouse, rat; applications: Immunofluorescence, Western Blot, Immunohistochemistry, Elisa; Validation: Manufacturer - https://www.scbt.com/p/syp-antibody-h-8?gclid=CjwKCAjwt52mBhB5EiwA05YKo4ePEOMyMIrqZzCaYKzlU_gx2A-wSZh0uQLJoBeVyQfX5oHfjp8eyxoCrcwQAvD_BwE)
anti-acetylated Tublin (Confirmed Species reactivity: bovine, frog, invertebrates, human, hamster, mouse, protista, pig, monkey, chicken, rat, plant; Applications: electron microscopy, immunohistochemistry, Immunofluorescence, western blot; Validation: Manufacturer - https://www.sigmaaldrich.com/US/en/product/sigma/t7451?gclid=CjwKCAjwt52mBhB5EiwA05YKowLtMY3KWAxOJZNqKiqoIubAK-awWidzUJv_yNP1GRyOl8sn0wqYNxoCs-UQAvD_BwE&gclsrc=aw.ds)
anti-ATP6V1G3 (Confirmed Species reactivity: Human; Applications: immunohistochemistry; Validation: Manufacturer - https://www.sigmaaldrich.com/US/en/product/sigma/hpa028701)
anti-Ki-67 (Confirmed Species reactivity: Mouse; Applications: immunocytochemistry; Validation: Manufacturer - https://www.thermofisher.com/antibody/product/Ki-67-Antibody-clone-SolA15-Monoclonal/14-5698-82)
anti-BSND (Confirmed Species reactivity: Mouse, Rat, Human; Applications: immunohistochemistry, western blot; Validation: Manufacturer - https://www.abcam.com/products/primary-antibodies/bsnd-antibody-epr14270-c-terminal-ab196017.html)
anti-FOXI1 (onfirmed Species reactivity: Human; Applications: Western Blot, Immunohistochemistry; Validation: Manufacturer - https://www.abcam.com/products/primary-antibodies/foxi1-antibody-ab20454.html)
anti-EGFP (Confirmed Species reactivity: Species independent; Applications: Western Blot, Immunohistochemistry; Validation: Manufacturer - https://www.abcam.com/products/primary-antibodies/gfp-antibody-ab13970.html)
anti-p63 (Confirmed Species reactivity: human; Applications: immunohistochemistry, Immunofluorescence, western blot; Validation: Manufacturer - https://www.stemcell.com/products/anti-human-p63-deltan-antibody-clone-poly6190.html)
anti-Muc5B (Confirmed Species reactivity: human; Applications: immunohistochemistry, Immunofluorescence; Validation: Manufacturer - https://www.sigmaaldrich.com/US/en/product/sigma/hpa008246?gclid=Cj0KCQjw2qKmBhCfARIsAFy8buIYdkjTn8ZEHkC66iUYoh13yOwm_A8Q8SUGxYNVfWEvD-Ya4q9GxAwaAkHjEALw_wcB&gclsrc=aw.ds)
anti-Muc5AC (Confirmed Species reactivity: Mouse, Rat, Human; Applications: immunohistochemistry, Immunofluorescence;

Validation: Manufacturer - https://www.abcam.com/products/primary-antibodies/mucin-5ac-antibody-45m1-ab3649.html)

Eukaryotic cell lines

Policy information about cell lines and Sex and Gender in Research

Cell line source(s)	Primary airway basal cells were derived from WT, FOXI1-CreERT2::ROSA-TG, FOXI1-KO, CFTR-KO, and FOXI1-CreERT2::CFTR-L/L ferrets.
Authentication	All primary airway basal cell lines were validated for genotype and performed from multiple donor as specified in the text.
Mycoplasma contamination	All primary cells tested negative for mycoplasma contamination prior to experimentation.
Commonly misidentified lines (See ICLAC register)	n/a

Animals and other research organisms

Policy information about studies involving animals; ARRIVE guidelines recommended for reporting animal research, and Sex and Gender in Research

Laboratory animals	Transgenic ferret models were used in this study including: WT ferrets, FOXI1-CreERT2::ROSA-TG ferrets, FOXI1-KO ferrets, CFTR-KO ferret, FOXI1-CreERT2::CFTRL/L ferrets and CFTR-G551D CF ferrets.<br>MCC studies were conducted on 5-6 month old FOXI1-CreERT2::CFTR-L/L ferrets.<br>MCC studies were conducted on 6-38 month old WT ferrets and FOXI1-KO ferrets.<br>MCC studies were conducted on 4-6 month old CFTR-G551D CF ferrets.<br>Tissue electrophysiology studies were conducted on 18-36 month old FOXI1-KO ferrets and age match WT ferrets.<br>Ionocyte Lineage tracing studies were conducted on 1-5 month old FOXI1-CreERT2::ROSA-TG ferrets.<br>Proximal airway stem cells differentiation studies were conducted on 1-5 month old FOXI1 KO ferrets strian and age match WT ferrets, FOXI1-CreERT2::ROSA-TG ferrets, FOXI1-CreERT2::CFTR-L/L ferrets, CFTR-KO ferret.<br>Ionocyte localization studies (trachea whole mount staining studies) were conducted on 6-12 month old WT ferret and CFTR-G551D CF ferrets.
Wild animals	This study did not involve wild animals.
Reporting on sex	Sex was not considered as a variable in our studies since it is hard to control equal distribution of sexes with transgenic ferrets.
Field-collected samples	This study did not involve samples collected from field.
Ethics oversight	All animal experimentation was approved by the Institutional Animal Care and Use Committee of the University of Iowa (Protocol: 0031945).

Note that full information on the approval of the study protocol must also be provided in the manuscript.

Flow Cytometry

Plots

Confirm that:

- [ ] The axis labels state the marker and fluorochrome used (e.g. CD4-FITC).
- [ ] The axis scales are clearly visible. Include numbers along axes only for bottom left plot of group (a 'group' is an analysis of identical markers).
- [ ] All plots are contour plots with outliers or pseudocolor plots.
- [x] A numerical value for number of cells or percentage (with statistics) is provided.

Methodology

Sample preparation	As indicated in the methods, fully differentiated ferret airway epithelia ALI cultures were dissociated using Accumax (Stem Cell Technologies) followed by DNase treatment. Cells were filtered through a 20 µM strainer and pelleted in 0.04% BSA PBS at 500g for 10 min. Nonviable dead cells were removed by using MACS Dead Cell Removal Kit following 10X Genomics recommendations (Document CG00039).
Instrument	Becton Dickinson Aria II

Software	BD FACSDiva 8.0.1 Software
Cell population abundance	Lineage traced pulmonary ionocytes (EGFP+) were <1% of total population.
Gating strategy	Cells were identified based on FSC and SSC gating. Tomato and EGFP positive epithelial cells were identified as positive based based on comparison to non-reporter ferret airway basal cells. Single cells were identifies based on forward scatter and forward pulse width.

☒ Tick this box to confirm that a figure exemplifying the gating strategy is provided in the Supplementary Information.
