# Supplementary material for: Transgenic ferret models define pulmonary ionocyte diversity and function
Source: Nature. 2023 Sep 20;621(7980):857–67. doi: 10.1038/s41586-023-06549-9 (PMC10533402; doi:10.1038/s41586-023-06549-9)
Supplement: Supplementary file 3 — Supplementary results and discussion. [file 41586_2023_6549_MOESM3_ESM.docx]

**Supplementary Results**

Experiments demonstrate that ionocytes directly transport CFTR-dependent anions from the ASL under fluid absorptive conditions, a finding consistent with slower fluid absorption rates in *FOXI1*-KO ALI cultures lacking ionocytes and *CFTR*-KO cultures lacking functional CFTR (Fig. 1d,e). However, *FOXI1*-KO ALI cultures and trachea also demonstrated a reduced ASL height at homeostasis like *CFTR*-KO airway epithelia (Fig. 1e,f; Extended Data Fig. 2e), suggesting that ionocytes might also be involved in anion secretion required for maintenance of ASL volume. Thus, we utilized the YFP sensor assay to evaluate halide movement from the basolateral surface into ionocytes. Initial attempts evaluated Cl**^–^**🡪I**^–^** exchange at the basolateral surface in equilibrated humidified *FOXI1*-Cre^ERT2^::YFP^H148Q/I152L^ ALI cultures and no YFP quenching was observed (Extended Data Fig. 3a). We reasoned that dehydration of the ASL might be required to promote ionocyte-mediated anion secretion and thus dehydrated the ASL with unhumidified air to simulate a reduced ASL height observed *CFTR*-KO cultures (Extended Data Fig. 2e,f). However, ASL dehydrated cultures also produced little YFP quenching following basolateral Cl**^–^**🡪I**^–^** exchange (Extended Data Fig. 3b). Notably, the addition of Cl**^–^** containing buffer to the apical surface promoted basolateral I**^–^** uptake by ionocytes (i.e., YFP quenching) and this process was inhibited by the basolateral Na-K-2Cl cotransporter-1 (NKCC1/*SLC12A2*) channel blocker bumetanide (Extended Data Fig. 3b,c), supporting the notion that ionocytes do participate in anion secretion. Furthermore, replacement of Cl**^–^** with gluconate in the apical fluid prevented basolateral I**^–^** uptake by ionocytes (Extended Data Fig. 3d), suggesting that Cl**^–^** may be necessary for proper channel function and/or electrical driving forces required for ionocyte-mediated salt and fluid secretion.

**Supplementary Discussion**

Here we applied conditional genetics and fate mapping in ferrets to dissect the biology and function of pulmonary ionocytes. Findings from this study demonstrate that pulmonary ionocytes transport the majority Cl**^–^** and HCO_3_**^–^** across proximal airway epithelia to regulate ASL volume, pH, and viscosity (Fig. 1). Each of these impaired functions in *FOXI1*-KO airway epithelia lacking ionocytes was similar to *CFTR*-KO ferret epithelia and well-described defects observed in CF human proximal airway epithelium^1,2^. Notably, ASL volume was regulated by independent phases of fluid absorption and secretion, and both phases were defective in airway epithelia lacking CFTR (*CFTR*-KO) or ionocytes (*FOXI1*-KO) (Fig. 1e). As predicted from these *in vitro* findings, *in vivo* tracheal MCC was similarly reduced in *FOXI1*-KO, *FOXI1*-CRE^ERT2^::*CFTR*^L/L^, and CF ferrets as compared to WT controls (Fig. 1i,j and Extended Data Fig. 3e,f). Similar studies in *Foxi1*-KO mouse tracheal epithelium have demonstrated compensatory channels in this species that counterbalance for the lack of ionocytes^3^. Consistent with the evolutionary divergence of this rare cell type in mice, pulmonary ionocyte transcriptomes were more closely conserved between human and ferret (Fig. 5).

Our single cell imaging studies using an ionocyte-specific halide YFP sensor and conditional *CFTR* deletion demonstrate that rare pulmonary ionocytes directly transport anions in a CFTR-dependent fashion, ruling out other non-cell autonomous mechanisms that could theoretically impact ion transport in *FOXI1*-KO epithelia lacking ionocytes (e.g., an ionocyte secreted factor that regulates ion transport by other cell types in a paracrine fashion). However, it remained unclear how such a rare cell type, which composes only ~1% of total cells in the proximal airway epithelium, can facilitate such a significant portion of anion transport and ASL volume regulation.

Key known channels involved in CFTR-dependent salt and fluid movement include the apical Na^+^ channel (ENaC) composed of three subunits (ENaCα,β,γ; *SCNN1A*, *SCNN1B*, *SCNN1G*), the basolateral Na^+^/K^+^/2Cl**^–^** symporter (NKCC1/*SLC12A2*), and the basolateral Na^+^/K^+^-ATPase composed of two subunits (*ATP1A1*, *ATP1B1*). ENaC and the Na^+^/K^+^-ATPase work in concert with CFTR to absorb NaCl in an apical🡪basolateral direction, whereas NKCC1 is required for NaCl secretion mediated by CFTR, both leading to water movement in the direction of NaCl flux. Important to both these processes are basolateral K^+^ channels, such as KCNQ1, which recycles K^+^ out of cells. We also found the outward rectifying basolateral CLCK Cl**^–^** channel subunit (*CLCNKA*) and its regulator (*BSND*) were differentially expressed at higher levels in ionocytes, and thus may function in concert with CFTR to move Cl**^–^** through ionocytes in an apical🡪basolateral direction. With the exception of *SCNN1B,* each of these channel-encoding genes were differentially expressed at higher levels in ionocytes and lends support for cell-autonomous movement of NaCl (Fig. 6a,b). Furthermore, six mitochondrial ATP synthase gene were differentially upregulated in ionocytes to support ATP or cAMP energy requirements of CFTR, KCNQ1, and Na^+^/K^+^-ATPase (*ATP1A1*, *ATP1B1*) (Supplementary Table 9). Alternatively, ionocytes-dependent NaCl and fluid movement could also be facilitated by gap junctional communication with neighboring luminal cells that establishing electrical driving forces (Fig. 6c,d). While this potential mechanism remains, we found only 5 connexin gap junctional genes (*GJB*) expressed in luminal cells (ciliated, secretory, goblet cells) and only a small percentage of ionocytes expressed very low levels of *GJBs* (Extended Data Fig. 5). Thus, we favor a cell-autonomous mechanism for ionocyte movement of NaCl required for fluid secretion and absorption.

While pulmonary ionocytes participate in regulating fluid and electrolyte balance in the ferret proximal airway epithelium, their abundance diminishes in the distal airways lacking glands and thus CFTR-expressing club secretory cells have been proposed to be the primary luminal cells regulating salt and fluid transport in the small airways^4^. In the proximal ferret airway epithelium, CFTR was also expressed at low levels in WT goblet (34%) and secretory cells (36%) (Supplementary Table 9, Extended Data Fig. 6i). Previous studies have demonstrated that proximal and distal human airway epithelium produce similar levels of CFTR-mediated current^4^; given that ionocytes are largely absent in the distal airways, the authors conclude that secretory cells (goblet and club) are responsible for most of the CFTR-mediated salt and water movement in the airways. This conclusion is contradictory to the 68-69% loss in CFTR-mediated anion transport in *FOXI1*-KO epithelia and suggests that proximal airway goblet cells may utilize CFTR for alternative functions in addition to regulating transepithelial anion and fluid movement. In this context, secretory cells in a number of cell systems express CFTR within the secretory granule where inhibition or absence of CFTR impairs mucin secretion^5-7^ and expansion^8,9^. The coupling of CFTR-mediated influx of Cl**^–^** and HCO_3_**^–^** into the secretory granule is thought to mediate membrane fusion, exocytosis, and proper unfolding of secreted mucins within the airway lumen. Differentiating the contributions of CFTR to transepithelial anion movement and mucin secretion in ferret airway secretory cells will require a conditional genetic model capable of deleting *CFTR* only in secretory cells.

Our studies demonstrate that basal cell specification of ionocyte is influenced by environmental changes in osmolarity. Indeed, ferret basal cells expressed channels known to be involved in cell volume regulation and sensing (*CLCN3, ATP1A1, ATP1B3, ATP1B1, TRPV4, TRPV2, AQP3, AQP4,* and *AQP5*) (Fig. 4g; Extended Data Fig. 5)^10-12^ and changes in cell volume have been shown to regulate stem cell fate decisions^13-15^. Given that hyperosmotic and hypoosmotic conditions imposed osmotic forces that dehydrated or over hydrated ASL, respectively (Fig. 4h), we speculate that the differential expansion or decline of ionocyte subtypes under these stress conditions (Fig. 4h,i) is mediated by an ASL volume sensing mechanism early following polarization. Such changes would predict that various ionocyte subtypes function differently to maintain ASL volume. For example, Type-A ionocytes appear best suited for fluid absorption since they are enriched in *CLCNKA*, *BSND*, *SCNN1A*, and *SCNN1B* (Supplementary Table 3). Thus, the induction of Type-A ionocytes under hypoosmotic stress (i.e., over hydrated ASL) is consistent with the expansion of ionocytes with a greater capacity to facilitate salt and fluid absorption (i.e., basolateral CLCNK outward rectifying chloride channel complex [CLCNKA / BSND], CFTR, and ENaC). Similarly, hypoosmotic stress induced Type-B and Type-A ionocytes which expression the highest levels *CFTR*, while hyperosmotic stress reduced CFTR-rich Type-B ionocytes but increased Type-C ionocytes that express the least amount of *CFTR*. This data supports the notion that apical membrane Cl**^–^** permeability is constrained by CFTR-expressing ionocytes and may be analogous to the inactivation of *CFTR* expression in fish gill ionocyte during sea water to fresh water transition^16^. Together with the finding that CF airways have greater numbers of ionocytes (Extended Data Fig. 7j,k; Extended Data Fig. 8c,d), this supports the concept that epithelial sensing mechanisms react to changes in osmolarity and/or ASL volume to control ionocyte numbers and phenotype.

Our single-cell atlas of ferret proximal tracheal epithelium provides an in-depth characterization of cellular phenotypes in a species that is widely used for respiratory disease research^17-20^. Species comparisons of rare cell type transcriptomes between human, ferret, and mouse demonstrate considerable similarities, but also reveal species-specific differences (Fig. 5). The identification of a rare cell progenitor with an overlapping tuft, PNEC, and ionocyte phenotype (Fig. 5a-f,h, Extended Data Fig. 12) is consistent with our *in vivo* and *in vitro* *FOXI1*-Cre fate mapping during neonatal airway development and basal cell differentiation, respectively (Extended Data Fig. 10 and 11). Type-C ionocytes, which have the ability replicate in ALI cultures, appear most closely related to this common rare cell progenitor (Extended Data Fig. 12) and thus appear to be a committed progenitor of Type-A and Type-B ionocytes. While *FOXI1* was not required for PNEC and tuft cell formation, the finding that *FOXI1*-KO epithelia contained significantly fewer PNECs and more tuft cells (Extended Data Fig. 6g; Extended Data Fig. 11g,h) suggests that the *FOXI1* gene may be functionally involved in basal cell specification events that determine rare cell frequency. This rare cell progenitor population was also enriched in genes (*KRT13* and *KRT4*) for the previously described Krt4**^+^**/Krt13**^+^** hillock-associated club cells of mouse trachea^3^. It remains unclear if the altered PNEC and tuft cell frequencies in *FOXI1*-KO ferrets impact the ionocyte-deficient phenotype.

**References**

1 Pezzulo, A. A. *et al.* Reduced airway surface pH impairs bacterial killing in the porcine cystic fibrosis lung. *Nature* **487**, 109-113 (2012)

2 Evans, T. I. *et al.* Glandular Proteome Identifies Antiprotease Cystatin C as a Critical Modulator of Airway Hydration and Clearance. *Am J Respir Cell Mol Biol* **54**, 469-481 (2016)

3 Montoro, D. T. *et al.* A revised airway epithelial hierarchy includes CFTR-expressing ionocytes. *Nature* **560**, 319-324 (2018)

4 Okuda, K. *et al.* Secretory Cells Dominate Airway CFTR Expression and Function in Human Airway Superficial Epithelia. *Am J Respir Crit Care Med* **203**, 1275-1289 (2021)

5 LeSimple, P. *et al.* Cystic fibrosis transmembrane conductance regulator is expressed in mucin granules from Calu-3 and primary human airway epithelial cells. *Am J Respir Cell Mol Biol* **49**, 511-516 (2013)

6 Kuver, R., Klinkspoor, J. H., Osborne, W. R. & Lee, S. P. Mucous granule exocytosis and CFTR expression in gallbladder epithelium. *Glycobiology* **10**, 149-157 (2000)

7 Thevenod, F. Ion channels in secretory granules of the pancreas and their role in exocytosis and release of secretory proteins. *Am J Physiol Cell Physiol* **283**, C651-672 (2002)

8 Gustafsson, J. K. *et al.* Bicarbonate and functional CFTR channel are required for proper mucin secretion and link cystic fibrosis with its mucus phenotype. *J Exp Med* **209**, 1263-1272 (2012)

9 Quinton, P. M. Both Ways at Once: Keeping Small Airways Clean. *Physiology (Bethesda)* **32**, 380-390 (2017)

10 Hoffmann, E. K., Lambert, I. H. & Pedersen, S. F. Physiology of cell volume regulation in vertebrates. *Physiol Rev* **89**, 193-277 (2009)

11 Liu, X. *et al.* A role for AQP5 in activation of TRPV4 by hypotonicity: concerted involvement of AQP5 and TRPV4 in regulation of cell volume recovery. *J Biol Chem* **281**, 15485-15495 (2006)

12 Sardini, A. *et al.* Cell volume regulation and swelling-activated chloride channels. *Biochim Biophys Acta* **1618**, 153-162 (2003)

13 Lee, H. P., Stowers, R. & Chaudhuri, O. Volume expansion and TRPV4 activation regulate stem cell fate in three-dimensional microenvironments. *Nat Commun* **10**, 529 (2019)

14 Willard, V. P. *et al.* Transient receptor potential vanilloid 4 as a regulator of induced pluripotent stem cell chondrogenesis. *Stem Cells* **39**, 1447-1456 (2021)

15 Guo, M. *et al.* Cell volume change through water efflux impacts cell stiffness and stem cell fate. *Proc Natl Acad Sci U S A* **114**, E8618-E8627 (2017)

16 Lam, S. H. *et al.* Differential transcriptomic analyses revealed genes and signaling pathways involved in iono-osmoregulation and cellular remodeling in the gills of euryhaline Mozambique tilapia, Oreochromis mossambicus. *BMC Genomics* **15**, 921 (2014)

17 He, N. *et al.* Ferret models of alpha-1 antitrypsin deficiency develop lung and liver disease. *JCI Insight* **7** (2022)

18 Sun, X. *et al.* In utero and postnatal VX-770 administration rescues multiorgan disease in a ferret model of cystic fibrosis. *Sci Transl Med* **11** (2019)

19 Enkirch, T. & von Messling, V. Ferret models of viral pathogenesis. *Virology* **479-480**, 259-270 (2015)

20 Peng, X. *et al.* The draft genome sequence of the ferret (Mustela putorius furo) facilitates study of human respiratory disease. *Nat Biotechnol* **32**, 1250-1255 (2014)
